# A New Metalic Compound for Casts

**Published:** 1852-04

**Authors:** 


A New Metalic Compound for Taking Casts.—Dr. Van Emon, has, we are told, succeeded in getting a compound of metals which is sufficiently hard for the male cast, and the peculiar and invaluable property of which is, that it does not shrink in cooling. We were informed by members of the dental class, that it gave a most perfect fac simile of the plaster model. Since the close of the session we have been so much occupied in fitting up a new office and other duties, that we have not as yet tried it.

It is prepared as follows: take five parts block tin and one of antimony. On preparing it the Dr. first takes equal parts of tin and antimony by weight, and melts together, then adds the remaining tin. If this answers the purpose we are told it does by all those who have tried it, we consider it invaluable.

